# Adaptation and Content Validation of the Belgian Winnipeg Assessment of Neonatal Nursing Needs Tool (BE‐WANNNT) for Neonatal Intensive Care Units

**DOI:** 10.1155/jonm/9557505

**Published:** 2026-07-23

**Authors:** Brenda Van Delft, Melissa Lauwerens, Manuela Bastanie, Ann Berghs, Ingrid Deroover, Dorien Evens, Geert Lingier, Anne Malcort, Marianne Peelman, Agna Preudhomme, Tony Waterschoot, Katrien Beeckman

**Affiliations:** ^1^ Universitair Ziekenhuis Brussel (UZ Brussel), Faculty of Medicine and Pharmacy, Nursing and Midwifery Group (NUMID), Primary Care (PRIM), Department of Neonatology, VINZ (Vlaamse Intensieve Neonatale Zorgen), Vrije Universiteit Brussel (VUB), Brussels, Belgium, vub.ac.be; ^2^ Department of Neonatology, Universitair Ziekenhuis Antwerpen (UZA), Edegem, Belgium; ^3^ Department of Neonatology, VINZ (Vlaamse Intensieve Neonatale Zorgen), Ziekenhuis Aan de Stroom (ZAS), Antwerp, Belgium; ^4^ Department of Neonatology, VINZ (Vlaamse Intensieve Neonatale Zorgen), Universitair Ziekenhuis Leuven (UZ Leuven), Leuven, Belgium; ^5^ Department of Neonatology, VINZ (Vlaamse Intensieve Neonatale Zorgen), Ziekenhuis Oost-Limburg (ZOL), Genk, Belgium; ^6^ Department of Neonatology, VINZ (Vlaamse Intensieve Neonatale Zorgen), Universitair Ziekenhuis Gent (UZ Gent), Gent, Belgium; ^7^ Groupe Santé CHC, Department of Neonatology, VINZ (Vlaamse Intensieve Neonatale Zorgen), Clinique CHC MontLégia, Liège, Belgium; ^8^ Department of Neonatology, VINZ (Vlaamse Intensieve Neonatale Zorgen), Algemeen Ziekenhuis Sint-Jan Brugge (AZ Sint-Jan), Bruges, Belgium

**Keywords:** NICU, workload

## Abstract

**Background:**

Continuous advancements in neonatal intensive care (NICU) have increased the complexity of care and the workload of nurses. Existing workload measurement instruments, such as the Winnipeg Assessment of Neonatal Nurses Needs Tool (WANNNT), are valuable but require adaptation to local healthcare settings.

**Objective:**

This study aimed to adapt and validate the WANNNT for the Belgian NICU context, resulting in the BE‐WANNNT.

**Methods:**

A 2‐year observational mixed‐methods study was conducted. First, the original WANNNT and WANNNT‐SC were translated and adapted to the Belgian context, incorporating corrected gestational age, updated respiratory modalities, and restructuring of indicators by physiological systems. Using the Policy Delphi method, an expert panel of NICU head nurses (*n* = 8) reviewed and refined 49 indicators across six consensus rounds. Subsequently, an online survey was distributed to NICU nurses and midwives (*n* = 76) to evaluate indicator clarity, relevance, and care intensity using a four‐point Likert scale. Qualitative data were analyzed thematically, while quantitative data were assessed with descriptive and inferential statistics.

**Results:**

The preliminary BE‐WANNNT was modified to reflect current Belgian NICU practices. Consensus was reached on the majority of indicators, with 45 of 49 achieving ≥ 75% agreement for clarity and relevance. Four indicators (e.g., low‐flow nasal cannula, neonatal abstinence syndrome < 8, cardiorespiratory monitoring, and saturation measurement) did not reach consensus but were retained after expert evaluation. Care intensity levels were confirmed for 38 indicators, while 11 required reconsideration, resulting in minor adjustments. The final BE‐WANNNT consists of six care levels and a contextually adapted and content validated set of indicators, enabling structured assessment of nursing workload in Belgian NICUs.

**Conclusion:**

The BE‐WANNNT is the first workload assessment tool specifically adapted for the Belgian NICU context. It provides a structured and contextually adapted framework to support workload assessment and evidence‐informed staffing discussions in neonatal intensive care. Future research is needed to evaluate inter‐rater reliability, additional psychometric properties, and the long‐term impact on workforce planning, nurse well‐being, and patient outcomes.

## 1. Introduction

Continuous advancements in healthcare result in increasing demand for healthcare systems to adapt to innovative technologies, treatments, and evolving definitions of quality and costs [1]. This is also the case in neonatal intensive care in Belgium, and there were just over 110.400 live births in Belgium in 2023 [2]. Pinpointing the number of neonatal intensive care unit (NICU) admissions is a challenge, yet it has been estimated that 12% of infants require neonatal intensive care for complex medical needs [3]. Patients’ care needs will always be of interest to nurses and other healthcare professionals, especially in the case of newborns in a fragile environment such as the NICU since the impact of health and wellbeing on adult life. As the demand in the NICU rises, personalized care of the infants and their parents has led to significant changes in the daily tasks of nurses. The organization of care has become increasingly critical as babies born as early as 23 weeks of gestation can now be treated, together with decreased length of stay for newborns due to technological advancements, i.e., discharged at home with tube feeding and/or respiratory support, discharged to rehabilitation center, and high‐tech equipment [4]. These changes result in a more complex logistical process and increase the nurse’s workload.

Nursing workload is a multifactorial phenomenon with a straightforward conceptual framework. This framework objective involves specific tasks that need to be completed. Nursing workload is defined as the “amount of performance required to carry out nursing activities,” often measured using resource‐based objective metrics, such as infant‐to‐nurse staffing ratios or patient acuity scores [5, 6]. There are different workload tools measurement systems (Northern Neonatal Network [NNN])–Patient Classification System [PCS]–NICU risk model …), but as a result of the innovations on the NICU ward, a more specific set of requirements is needed, and for this reason, the previous tools are outdated and no longer applicable to the current neonatal care setting. In 2015, the NICU department of UMC Utrecht developed her own workload tool “Neonatal Acuity‐based Patient Scoring System” (NAPSS). This workload tool is developed by combining the Winnipeg Assessment of Neonatal Nurses Needs Tool (WANNNT) and the PCS‐tools. This workload tool is developed to measure the nurse–patient ratio and includes the seriousness of the case and the nurse’s workload. However, up till now, the NAPSS‐tool [7] remained a theoretical exercise without implementation in practice.

The WANNNT, validated by Sawatzky Dickson and Bodnaryk [8] and the adapted WANNNT‐SC neonatal surgical intensive care unit in 2019 [9], provides a more overall approach. The WANNNT is an instrument designed to assess the care complexity of newborns admitted to the NICU. It was selected because the tool reflects the technological and clinical advancements that have occurred in neonatal intensive care. The WANNNT consists of a series of clear and visible patient indicators. Each indicator in the tool is assigned to a level of care, and a predetermined number of full‐time equivalents (FTE) is linked to each level, reflecting the care needs of the patients [8, 9].

The WANNNT determines the number of nurses needed for specific patients during the neonatal period. It is developed to select the most apparent and visible patient indicators while considering the patient’s needs for specific observations and monitoring by the nurse. Although the tool is not task‐oriented, a higher care level will reflect a higher workload. The WANNNT is a dynamic workload scale; indicators can be adjusted according to the current needs and standards of neonatal care [8].

In the context of optimizing workforce efficiency while ensuring personalized and high‐quality care, the implementation of a robust workload assessment tool is essential. NICU’s in Belgium are actively seeking such instruments.

The aim of this paper is to adapt the WANNNT tool for use in the Belgian NICU context. The second objective is to validate the WANNNT by a panel of experts and the user group of nurses and midwives active on NICU.

## 2. Materials and Methods

### 2.1. Type of Study

This was a 2‐year observational and descriptive study employing both qualitative and quantitative methodologies. Documentary review and fieldwork were utilized to define indicators for Belgium NICUs. A policy Delphi was chosen as a method to define consensus on the content and formulation of the indicators. Ethical approval was obtained from the Ethical Committee of UZ Brussel (BUN: 1432021000454).

### 2.2. Methods

This study followed the framework proposed by Dickson and Bonaryck [8], who recommend evaluating each indicator in the WANNNT in terms of clarity, relevance, and assigned care intensity using an expert panel of senior nurses. In the present, this approach was extended by involving a broader population of nurses and midwives working in Flemish NICUs in Belgium, in order to enhance contextual relevance and generalizability. Nurses without direct patient contact were excluded. Informed consent was obtained from all participants prior to participation.

### 2.3. Development of the Initial BE‐WANNNT

In the first phase, both the original WANNNT developed by Dickson and Bonaryck [8] and the later adapted version for WANNNT‐SC by Hart et al. [9] were used as foundational frameworks for the development of the BE‐WANNNT. Authorization for translation and adaptation was obtained from the original authors.

The original WANNNT was translated into Dutch by a native Dutch‐speaking nurse fluent in English. Subsequently, the study investigator conducted a comparative content analysis of both tools in relation to the Belgian neonatal care context. Based on this analysis, an initial draft of the BE‐WANNNT was developed. Indicators from both instruments were reviewed, merged, adapted, or excluded depending on their relevance to current clinical practice. Particular attention was given to ensuring applicability across a heterogeneous NICU population, including both medical and surgical patients.

Key modifications included the incorporation of corrected gestational age, restructuring of indicators according to physiological systems (monitoring, nursing care…), and alignment with national standards. Surgical and other specialized indicators (e.g., neurocritical, cardiovascular, and hemodynamic care) were selectively integrated where relevant, while avoiding overspecification to maintain the tool’s applicability across different patient subgroups.

This approach reflects the use of WANNNT as a dynamic and adaptable instrument, allowing the integration of elements from both general and specialized versions into a context‐specific tool prior to formal validation.

### 2.4. Content Validity by Experts Via Policy Delphi Method (Preliminary BE‐WANNNT)

Content validity of preliminary BE‐WANNNT was assessed using a Policy Delphi method, which is designed to systematically collect and structure expert opinions on complex healthcare issues [10–12]. A panel of expert head nurses from the Flemish NICU network (VINZ) was purposively recruited based on their clinical expertise and familiarity with neonatal workload. The initial draft tool, comprising 49 indicators, was evaluated across six iterative Delphi rounds. The first four rounds focused on the qualitative evaluation of indicators, during which panel members rated clarity, relevance, and care intensity and provided qualitative feedback. After each round, responses were synthesized and used to refine the indicators. Following the large‐scale quantitative survey, two additional Delphi rounds were conducted with the same expert panel to review and interpret the quantitative findings. These rounds allowed for integration of survey results with expert judgment and, where necessary, further refinement of the indicators. Indicators for which ≥ 75% agreement was achieved were retained. Indicators with 60%–74% agreement were revised and re‐evaluated in subsequent rounds, while those scoring below 60% were excluded. The Delphi process consisted of six rounds, during which panel members provided both ratings and qualitative feedback.

### 2.5. Content Validity and Estimation of Care Intensity in a Large Sample of Care Providers by Online Questionnaire

Following the Delphi rounds, a structured online questionnaire [13] was developed to validate the indicators in a broader sample. The survey was distributed via Qualtrics XM to nurses and midwives across all Flemish NICUs. No formal sample size calculation was performed, as Delphi studies rely on expert consensus rather than statistical power calculations. Recommended Delphi panel sizes vary in the literature and depend on the intended purpose, expertise, and heterogeneity of the panel members [12, 14]. In this study, inclusion of nurses and midwives from multiple Flemish NICUs provided sufficient professional diversity and contextual representation to support applicability within the NICU environment.

Participants evaluated each indicator on clarity, relevance, and estimated care intensity level. Responses were collected using a four‐point Likert scale (1 = *strongly disagree* to 4 = *strongly agree*). The questionnaire included sections on professional background, clarity of indicators, relevance of indicators, and estimation of care intensity. Each section included an open‐ended comment field to capture qualitative feedback. Indicators with < 75% agreement in this phase were marked for re‐evaluation by the expert panel.

In parallel, the expert panel assigned fractional care values to each indicator to reflect required nursing workload, based on the original WANNNT methodology [8]. Each level corresponds to a fractional nurse‐to‐patient workload, with Level 1 reflecting the lowest intensity (e.g., 0.3 FTE per infant) and Level 6 representing the highest (e.g., 1.0 FTE per infant). The adapted BE‐WANNNT incorporated these estimations and included additional domains, such as neurocritical, cardiovascular/hemodynamic, and surgical care, based on the current NICU practice. These modifications served as the foundation for the subsequent validation process.

### 2.6. Implementation of the BE‐WANNNT in the Flemish NICUs

Following the development and validation phases, the BE‐WANNNT was introduced into clinical practice within participating NICUs. To support consistent application, standardized training sessions were provided by the study investigator to designated users, including head nurses, deputy head nurses, and care coordinators. These sessions focused on the interpretation of indicators, scoring procedures, and practical use of the tool in daily workload assessment.

### 2.7. Data Analyses

#### 2.7.1. Qualitative Procedure: Policy Delphi Method

**FIGURE 1:**
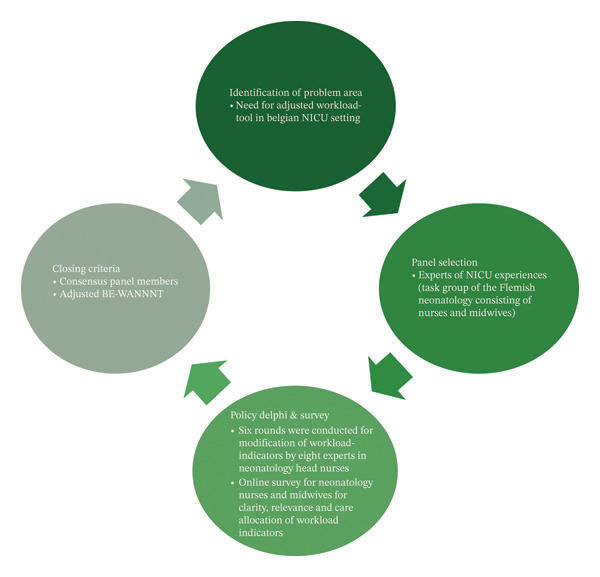
Overview of the Policy Delphi process used for refinement of the BE‐WANNNT.

To support the development and refinement of the BE‐WANNNT, a qualitative Policy Delphi method was used. This approach enabled the structured gathering of expert opinions over multiple iterative rounds to reach consensus on the workload indicators for neonatal intensive care. Data were collected from a selected panel of experts in neonatal care, all of whom were head nurses or had extensive clinical experience within Flemish NICUs (Figure 1).

The process began with the identification of the problem area, namely, the need to update an existing workload instrument to reflect the current neonatal care context. Subsequently, a panel was selected from the Flemish Neonatology Task Group, composed of nurses and midwives with frontline NICU expertise. Over six Delphi rounds, indicators were reviewed, discussed, and modified in terms of their formulation and relevance to care intensity. Qualitative data emerging from these rounds were analyzed using a framework approach, a widely used method for thematic analysis in applied health research [12–15]. This approach facilitated systematic coding and categorization of expert feedback, enabling identification of themes related to clarity, relevance, and feasibility of each indicator. The coding framework was both informed by the structure of the BE‐WANNNT and refined inductively based on the panel’s input across rounds.

The Delphi process was concluded when a sufficient level of agreement among panel members was reached, as defined by pre‐established consensus criteria. This final stage led to the adjustment and validation of the BE‐WANNNT in its current form.

#### 2.7.2. Quantitative Procedure: Online Questionnaire

The results of the quantitative data, collected from the survey, were subjected to descriptive analysis (frequency, mean, and standard deviation), and inferential analysis using SPSS Version 29. Based on the research by Pinto, Santos and Pires [16], consensus for clarity, relevance, and care allocation was determined using a median of ≥ 3 and a cumulative frequency of ≥ 75% in the four‐point Likert scale scores 3 and 4. Given the ordinal nature of the measurement, the variables were analyzed at the ordinal level, and the median was calculated as a measure of central tendency, distinguishing the lowest 50% of the values from the highest 50% [17].

## 3. Results

### 3.1. Adaptation of Indicator Descriptions to the Belgian NICU Context: BE‐WANNNT Preliminary

Following the translation of the original and adapted surgical WANNNT [8, 9], into the initial BE‐WANNNT, the indicator descriptions were adapted to the clinical context of Belgian NICUs. This adaptation was guided by six structured focus group meetings, each lasting approximately 2 hours, involving a panel of NICU experts. Through an iterative qualitative process, consensus was reached on various contextual modifications to ensure the tool’s relevance and usability in Belgium.

Several terminological and structural changes were implemented. For instance, the label “treatments” was revised to “nursing care.” This change reflects conceptual differences in interpretation: In the Belgian context, “treatments” are typically understood as medical interventions aimed at curing disease (often pharmacological), whereas “nursing care” encompasses supportive procedures, monitoring, and the clinical judgment required to manage patient responses and ensure continuity of care.

Additionally, in the “respiratory” category, respiratory modalities currently in use in Belgian NICUs were added to better reflect contemporary clinical practice (see Appendix 1). Broadly, the classification of corrected gestational age was updated in accordance with recent international standards. This was particularly important given the evolution of neonatal care for extremely premature infants (< 28 weeks’ gestation) since the original development of the WANNNT tool in the late 20th century [18]. These initial modifications formed the basis for the preliminary version of the BE‐WANNNT, which was subsequently evaluated in Delphi and survey phases.

### 3.2. Validation of the BE‐WANNNT by Experts: Policy Delphi Method

Six focus group sessions were conducted, each lasting 2 h using semistructured discussion guides. The expert panel consisted of eight head nurses from the Flemish Neonatal Intensive Care Network (VINZ), all with substantial clinical experience in neonatology. During these sessions, each workload level and its associated indicators within the preliminary BE‐WANNNT were systematically reviewed and discussed in depth. Across the six workload levels, most indicators, particularly those representing common nursing care activities in neonatology, were rated as essential or important by more than 80% of panel members. These results indicate a high level of consensus on the core components of the tool.

Several definitional adjustments were made to refine the metrics compared to the preliminary version of WANNNT. Nevertheless, the FTE allocations per workload level, as originally defined by the WANNNT authors, were retained. Given the minimal structural changes to the workload levels, the original FTE allocations were unanimously accepted by the panel. The final version of the BE‐WANNNT, integrating both qualitative and quantitative input from the expert panel, is presented in Appendix 1.

### 3.3. Validation of the BE‐WANNNT

#### 3.3.1. Participants

Data were obtained from 76 nurses and midwives (Table 1). Most of the nurses and midwives (47 (61.8%) had at least more than 10 years’ experience in the NICU.

**TABLE 1:** Work experience in the NICU (*N* = 76).

Work experience in years	*N* (%)
< 1	2 (2.6)
1–5	18 (23.7)
6–10	9 (11.8)
> 10	47 (61.8)

#### 3.3.2. Clarity of the Indicators

The clarity of the indicators described in the BE‐WANNNT had a median between 3 and 4 which means that > 75% of the participants agreed with the clarity of the indicators. Nine of the indicators did not meet the conditions and scored less than 75%. These indicators were taken back to the Delphi group for discussion and agreement.

#### 3.3.3. Relevance of the Indicators

Of the 49 indicators proposed, 45 were rated as relevant by more than 75% of participants in the large‐scale survey. Four indicators—low‐flow nasal cannula (LFNC), neonatal abstinence syndrome (NAS) without clinical signs (Finnegan score < 8), cardiorespiratory monitoring, and saturation measurement—did not reach and were returned to the expert panel for re‐evaluation within the Delphi process. Survey participants expressed concerns about the inclusion of these indicators, suggesting that they may not warrant a specific workload classification within the BE‐WANNNT. However, after in‐depth discussion and critical reflection, the Delphi panel decided to retain all four indicators in the final tool. This decision was based on a consensus that their clinical relevance and potential impact on nursing workload were insufficiently recognized in the quantitative phase, and that omission would risk underestimating care complexity in certain neonatal subpopulations.

#### 3.3.4. Validation of Care Intensity Levels Assigned in the BE‐WANNNT

Each care activity in the BE‐WANNNT was assigned an initial care level (1–6), reflecting estimated nursing workload. In this part of the validation process, participants were asked to assess whether the proposed care level per indicator was appropriate. For 38 out of the 49 indicators, the assigned care level was confirmed. For the remaining 11 indicators (as shown in Table 2), participants proposed an alternative care level.

**TABLE 2:** Proposed care level by respondents of the survey (*N* = 76) compared to the BE‐WANNNT proposed care level.

Indicators	Care level BE‐WANNNT	Frequency of proposed care level (*N* (%))					
		Level 1	Level 2	Level 3	Level 4	Level 5	Level 6
Breast/bottle/tube feeding guidance	1		30 (85.7)	5 (14.3)			
Healthy newborn with social problems	1		16 (80.0)	4 (20.0)			
Hyperbilirubinemia (10% below the change limit) within intensive phototherapy	3		17 (100.0)				
Newborn < 32 weeks’ gestation within the first week of live	4			5 (14.7)	29 (85.3)		
Invasive monitoring (blood pressure, venous pressure,…)	4		1 (3.1)	3 (9.4)	28 (87.5)		
Vasopressors phased out with stable blood pressure	4		2 (10.5)	16 (84.2)		1 (5.3)	
≥ 3 adjustments of IV‐fluid/TPN	4		3 (17.6)	14 (82.4)			
NIV‐NAVA, NIPPV, BiPAP with respiratory stimulation medication (except caffeine)	4		2 (13.3)	13 (86.7)			
CFM and/or NIRS	5		2 (4.8)	18 (42.9)	22 (52.4)		
Cardiopathy with prostaglandines	5			6 (37.5)	9 (56.3)		1 (6.3)
Newborn < 29 weeks’ gestation within the first week of live	5			4 (25.0)	12 (75.0)		
External ventricular drain	5			7 (46.7)	8 (53.3)		

*Note:* BiPAP: bilevel positive airway pressure; IV: intravenous; *N*: number; NIPPV: noninvasive positive pressure ventilation; NIRS: near infrared spectroscopy; NIV‐NAVA: noninvasive neurally adjusted ventilatory assist; BE‐WANNNT: Belgian Winnipeg Assessment of Neonatal Nursing Needs.

Abbreviations: CFM, cerebral function monitoring; TPN, total parenteral nutrition.

For the indicators “breast/bottle/tube feeding guidance” and “healthy newborn with social problems,” respectively 85.7% and 80.0% of participants suggested a higher care level (Level 2 instead of Level 1). Despite this, the proposed change was not adopted because the indicator definitions did not meet the threshold for systematic revision, and the expert panel deemed the current classification as sufficiently reflective of workload.

Several indicators originally classified as Level 4 or 5 were suggested to be shifted down by one or two levels. For example, “hyperbilirubinemia within intensive phototherapy” showed unanimous agreement (100%) to lower the level from 3 to 2. Similar patterns were observed for indicators such as “vasopressors phased out with stable blood pressure” and “invasive monitoring,” where most respondents proposed lower levels. In contrast, for the indicator “extern ventricular drain,” more than half (53.3%) suggested a lower level (from 5 to 4), but a significant minority (46.7%) retained the original level, indicating a split opinion.

The distribution of suggested care levels provides important insight into how frontline care providers perceive workload intensity in NICU care. However, based on both the strength of the Delphi methodology and expert consensus, only limited revisions were made. The original care levels were retained unless a clear majority (≥ 75%) and expert agreement supported adjustment.

## 4. Discussion

Neonatal nurses fulfill a critical role within the NICU, engaging in acute care, outpatient follow‐up, engaging in the day‐to‐day management of infants, advanced procedures, delivery room resuscitation, and neonatal transport [18]. Their workload has significant implications not only for their well‐being but also for the quality and safety of neonatal care delivery [5]. Accurate workload assessment is therefore essential to support effective staffing and safe care.

The WANNNT offers a structured method to determine nursing staff needs based on patient acuity. Our study aimed to contextualize this tool to the Belgian context. Through expert consultation using the Policy Delphi method and validation with a broad cohort of neonatal nurses, we developed the BE‐WANNNT: a practice‐ready version tailored to the Flemish NICU setting. While the original WANNNT, developed in the United States, provided a solid foundation for adaptation, both the original version and the later surgical adaptation (WANNNT‐SC) were reviewed during the developmental phase of this study. Rather than adopting one version exclusively, we applied an integrative approach in which relevant indicators from both instruments were critically appraised and selectively incorporated. This allowed the development of a context‐specific tool that reflects the heterogeneity of the NICU population, including both medical and surgical patients, while avoiding overspecification toward a single subpopulation.

Many existing workload tools are outdated and lack contextual fit. In this study, the WANNNT was approached as a dynamic and adaptable framework, enabling alignment with the Belgian care landscape and contemporary NICU practices. This flexibility was essential to ensure applicability across different organizational structures and patient profiles.

A key strength of our study is the integration of qualitative and quantitative methodologies to ensure comprehensive contextualization. Qualitative methods, such as focus groups and expert panels, allowed us to align the tool with actual clinical practices, enhancing its acceptability and utility. In addition, the iterative Delphi process, including follow‐up rounds after the quantitative survey, enabled the integration of broader clinical output with expert judgment, strengthening the robustness of the final tool. Previous research underscores that involving nursing professionals in validation improves applicability and commitment to implementation [19, 20]. In complex healthcare environments such as NICUs, qualitative insights are indispensable to complement quantitative data and capture the full scope of nursing workload [19].

While the majority of indicators (over 75%) were perceived as clear and relevant by the participants, approximately 25% did not achieve this threshold. The survey did not provide specific qualitative feedback explaining this discrepancy. Nonetheless, the expert panel chose to retain four indicators that did not achieve full consensus, based on clinical reasoning and the recognition that these activities, while often viewed as routines, significantly contribute to nursing workload. Rationale for retaining the indicators: (1) LFNC is a commonly used noninvasive respiratory support modality in infants with chronic lung disease (CLD) and is frequently applied to facilitate earlier discharge. Although the oxygen flow remains < 2 L/min with a fraction of inspired oxygen (FiO_2_) up to 100%, the nursing workload remains considerable due to continuous monitoring, adherence to weaning protocols, and extensive parental education. These factors justify their classification as a workload‐intensive care activity [21, 22]. (2) NAS without clinical signs (Finnegan score < 8): even in the absence of overt withdrawal symptoms, infants with suspected NAS require vigilant observation, individualized care, and regular standardized assessments (e.g., Finnegan or Eat, Sleep, Console). These structured evaluations demand sustained nursing attention, justifying inclusion in the workload tool [23]. (3) Cardiorespiratory Monitoring and Saturation Measurement: Although often perceived as standard practice, these tasks require technical precision and constant vigilance, particularly in high‐risk or unstable neonates. Responsibilities include sensor placement, alarm management, trend interpretation, and documentation. Omitting such indicators would underestimate the complexity and cognitive demands involved in routine monitoring.

The inclusion of these four indicators highlights the importance of combining empirical data with expert clinical judgment. It also underscores that perceived simplicity of a task does not necessarily equate to low workload. Such insights are essential when designing tools intended to capture the multifaceted nature of nursing workload in NICUs.

The results and subsequent adaptations of the BE‐WANNNT revealed that each NICU in Flanders operates under its own clinical and organizational nursing policies. This tool will make it possible to identify and compare these differences in practice. Although the BE‐WANNNT was developed in collaboration with all Flemish NICUs, NICUs in the French‐speaking regions of Belgium also follow distinct care policies. Therefore, further research involving NICUs across all regions of Belgium is recommended to support the development of a unified national workload tool.

The complex and demanding nature of neonatal care is a well‐established contributor to elevate stress levels among NICU nurses [24]. Improved workload measurement and management could lead to enhanced job satisfaction and staff retention in these settings. However, the results of this study may not be generalizable to NICUs in other countries. Nevertheless, the tool offers a robust and transferable framework that can be adapted to other national contexts through a similar structured and participatory process as employed in this study.

### 4.1. Strengths and Limitations

A major strength of this study lies in the structured, participatory development of a validated workload assessment tool specifically for the Flemish NICU context. To our knowledge, this is the first Belgian (and possibly international) study to involve a sizable sample of neonatal nurses and midwives (*n* = 76) in evaluating the clarity and relevance of workload indicators. This cocreative approach strengthens the clinical relevance and real‐world applicability of the tool.

The response rate of 17%, based on an estimated target population of 450 NICU professionals in Flanders, is close to the generally accepted range of 20%–30% for survey‐based studies in healthcare settings [25]. This response level reflects substantial professional engagement with the topic.

However, an important limitation is that the inter‐rater reliability of the BE‐WANNNT was not assessed. In the current implementation, workload assessments are primarily performed by designated staff members (head nurses, deputy head nurses, or care coordinators), who are familiar with the tool and received standardized training. This may contribute to a more consistent interpretation and application of the instrument in daily practice. Nevertheless, formal evaluation of inter‐rater reliability remains necessary and should be addressed in future research. Future studies should evaluate the consistency of tool application across raters and settings using statistical measures such as Cohen’s Kappa, which accounts for agreement by chance and offers a more rigorous assessment than simple percent agreement [26].

Furthermore, while content validity was addressed through expert consultation and user feedback, other psychometric properties such as construct validity and responsiveness over time were not examined in this study. Longitudinal implementation and outcome‐based evaluation will be essential to determine the tool’s long‐term impact on staffing decisions, job satisfaction, and patient outcomes. In addition, future research should include a systematic comparison with existing workload instruments, including specialized neonatal tools, to further strengthen the external validity of the BE‐WANNNT.

## 5. Conclusion

The original WANNNT was successfully adapted to the Belgian NICU context, resulting in the BE‐WANNNT. This instrument comprises six levels of care intensity and a set of contextually validated nursing care indicators reflecting contemporary neonatal practice. The BE‐WANNNT can be considered a useful and practice‐oriented tool for assessing nursing workload within the Belgian setting and may support informed nurse staffing decisions at both patient and unit levels. However, given the developmental and context‐specific nature of this study, further research is warranted. In particular, future studies should evaluate the reliability and practical applicability of the tool in daily clinical practice. In addition, a systematic comparison between the BE‐WANNNT, the original WANNNT, and its surgical adaptation (WANNNT‐SC), as well as other existing neonatal workload instruments, is recommended to further establish its validity and generalizability. The implementation of a structured and evidence‐informed workload tool remains essential to support safe, high‐quality neonatal care and to mitigate nursing workload.

## Funding

There was no funding.

## Conflicts of Interest

The authors declare no conflicts of interest.

## Data Availability

All data are available under reasonable request to the corresponding author.

## Appendix

Table A1 and A2

**TABLE A1:** Revised BE‐WANNNT‐scale for Belgian NICU’s.

Level	Category	Indicators
1. 0.25	Monitoring	• Postnatal care of a healthy term/dysmature newborn with or without phototherapy
		• Healthy newborn with social problems e.g., adoption, mother in ICU…

2. 0.30	Monitoring	• Hyperbilirubinemia (10% below the exchange limit) with intensive phototherapy
		• Observation of glucose, Pavlic harness, shoulder distortion, clavicula#, plaster
	Neuro	• Neonatal abstinence syndrome without clinical signs (Finnegan score < 8)
	Respiratory	• LFNC < 2 L/min
	Nutrition/IV	• PI on heparin lock or with IV‐solution (glucose or TPN)
		• Tube feeding duodenal or gastric or gastrostomy (continuous or fractionated)
		• Breast/bottle/tube feeding guidance
		• EFS (until end of procedure)
		• Wound care (> 2 per day, excluding protective crème/ointment to prevent dermatitis)
	Nursing care	• Stomy care with or without re‐feeding

3. 0.50	Monitoring	• Newborns between 30^0/7^ and 32^0/7^ gestational age during the first week of life
		• > 2/hour cardiorespiratory alarms with > 1 intervention(s) (with tactile stimulation)
		• > 4 glucose controls/6 hours and/or supplementary feeding or starting glucose in babies of IDM’s
		• Invasive pressure monitoring (blood pressure, CVP…)
	Neuro	• Neonatal abstinence syndrome with withdrawal symptoms (Finnegan score > 8)
	Respiratory cardiovascular	• CPAP/High HFNC > 2l/min./NIV‐NAVA/NIPPV/tracheostomy
		• Vasopressors being tapered with stable blood pressures.
	Nutrition/IV	• PICC/CVC/Art. catheter/UVC/Broviac
		• > 3 adjustments of IV‐fluids/TPN per 24 hours
	Nursing care	• Adjust > 2/6u nutritional policy, CGA, …
		• One‐time bladder probe (at least twice a day) for example for spina bifida

4. 0.70	Monitoring	• Newborn between 28 ^1/7^ and 29 ^6/7^ gestational age during first week of life
		• > 4/hours cardiorespiratory alarms with intervention with mask and bag
	Neuro	• CFM and/or NIRS
		• Cooling
		• Convulsions
		• External ventricular drain
		• Mechanical ventilation
	Respiratory	• NIV NAVA–NIPPV–BiPAP with respiratory stimulation medication (excluding caffeine)
		• Chest drain (1)
	Cardiovascular Nursing care	• Cardiopathy with prostaglandins
		• > 3 Stoma changes per day with or without refeeding
		• > 3 Wound care dressings per day

5. 1.00	Monitoring	• Newborn between 26^0/7^ and 28^0/7^ gestational age during first week of life
		• End of life care/comfort therapy in assisted dying
	Respiratory	• Mechanical ventilation (PRVC/NAVA/HFO) with > 2 blood gas checks and adjustments
		• NO‐therapy
		• More than 1 chest tube in situ
	Cardiovascular	• 2 of more Inotropes with unstable blood pressures
	Nefro	• Peritoneale dialyses or hemodialyses

6. 1.50	Monitoring	• Newborns < 25^6/7^ gestational age in the first 3 weeks of life
		• Surgical procedures in the NICU
	Cardiovascular	• Hemodynamically unstable neonate with > 2 inotropies +dos adjustments (> 1 adjustments per hour)
		• Postoperative cardio‐surgery (excluding PDA) during the first 48 hours.
	IV	• Newborn with DIC
	Nursing care	• Newborn with epidermis bullosa or collodion

*Note:* BiPAP: bilevel positive airway pressure; IDM: insulin‐dependent diabetes mellitus; NIPPV: noninvasive positive pressure ventilation; NIRS: near infrared saturation; NIV NAVA: noninvasive ventilation neurally adjusted ventilatory assist.

Abbreviations: CFM, cerebral function monitoring; CGA, continuous gastric aspiration; CPAP, continuous positive airway pressure; CVC, central venous catheter; CVP, central venous pressure; DIC, diffuse intravascular coagulation; EFS, early feeding skills; HFNC, high flow nasal canula; HFO, high frequency oscillation; ICU, intensive care unit; IV, intravenous; LFNC, low flow nasal cannula; NICU, neonatal intensive care unit; NO, nitric oxide; PDA, persistent ductus arteriosus; PI, peripheral infuse; PICC, peripherally inserted central catheter; PRVC, pressure release volume control; TPN, total parenteral nutrition; UVC, umbilical venous catheter.

**TABLE A2:** BE‐WANNNT‐scale.



Abbreviations: NICU, neonatal intensive care unit; NIDCAP, Newborn Individualized Development Care and Assessment Program.
